# Emerging Comorbidities in Inflammatory Bowel Disease: Eating Disorders, Alcohol and Narcotics Misuse

**DOI:** 10.3390/jcm10194623

**Published:** 2021-10-08

**Authors:** Paweł Kuźnicki, Katarzyna Neubauer

**Affiliations:** 1 Department of Gastroenterology and Hepatology, Wroclaw Medical University, Borowska 213, 50-556 Wroclaw, Poland; pawel.kuznicki@student.umed.wroc.pl; 2 Department of Gastroenterology and Hepatology, Division of Dietetics, Wroclaw Medical University, Borowska 213, 50-556 Wroclaw, Poland

**Keywords:** inflammatory bowel disease, ulcerative colitis, Crohn’s disease, alcohol, narcotics, cannabinoids, substance use disorders, eating disorders, anorexia, bulimia

## Abstract

Inflammatory bowel disease (IBD) is a chronic and potentially devastating condition of the digestive tract which is exemplified by increasing prevalence worldwide, byzantine pathogenesis with a poorly defined role of the environmental factors, and complex clinical demonstration. As a systemic disease, IBD may progress with a wide spectrum of extraintestinal manifestations (EMs) and comorbidities affecting different organs and systems, from anaemia, undernutrition, and cancer to those which are often neglected like anxiety and depression. Evolving IBD epidemiology and changing environment are reflected by an expanding list of IBD-related comorbidities. In contrast to the well-established role of smoking the connection between alcohol and IBD is still debatable on many levels, from pathogenesis to complications. Furthermore, little is known about narcotics use in IBD patients, even if there are obvious factors that may predispose them to narcotics as well as alcohol misuse. Last but not least, the question arises what is the prevalence of eating disorders in IBD. In our paper, we aimed to discuss the current knowledge on alcohol and drugs misuse and eating disorders as emerging extraintestinal comorbidities in IBD.

## 1. Introduction

At the beginning of the 21st century, we are witnessing a change in paradigms related to inflammatory bowel disease (IBD) and chronic conditions of the gastrointestinal tract represented by two major forms: ulcerative colitis (UC) and Crohn’s disease (CD). IBD typically develops in young adults, has a relapsing course with periods of flares and remissions, manifests up from the subtype, location, and activity of disease with bloody diarrhoea, abdominal pain, and unintended body weight loss. Additionally, IBD is associated with a wide spectrum of extraintestinal manifestations (Ems) [1,2]. IBD has complex nature and its etiopathogenesis is only partly revealed, with special attention put recently on environmental triggers and gut microbiota [3]. Despite the huge progress in the diagnostic techniques, the time from the first symptoms to final diagnosis remains long, which can negatively influence the disease outcome [4,5]. There is no single “golden diagnostic test” available and the establishment of IBD diagnosis relies on the combination of clinical symptoms, endoscopic, and histopathological findings, supported by imaging and laboratory tests.

The occurrence of IBD is increasing worldwide and they are no longer diseases of the Western countries [6]. Based on data from the Global Burden of Diseases, Injuries, and Risk Factors Study (GBD) 2017, between 1990 and 2017 the number of individuals with IBD increased from 3.7 million to more than 6.8 million (3.9 million females and nearly 3 million males) globally. Furthermore, the total number of IBD-related deaths increased by 67% from 1990 to 2017, from 23,000 to 38,000. The highest age-standardised prevalence rate globally was noted in the United States (464.5 per 100,000 population), whereas among European countries the highest prevalence was in the United Kingdom (449.6 per 100,000 population) [7].

It was repetitively demonstrated that IBD is associated with lower Health-Related Quality of Life (HRQoL) which is an important measure of the global influence of disease on a person’s physical, mental and emotional well-being. IBD affects interpersonal relationships, life activities, social participation, and mental well-being [8,9,10]. Psychological support does not belong to the standard care of patients with IBD although it is suggested that psychotherapeutic interventions can improve the quality of life of individuals with IBD [11]. Furthermore, IBD may compromise patients’ workability and impact other parameters of work impairment, such as absenteeism, presenteeism, activity, and productivity loss [12]. Moreover, work disability is linked directly with the indirect costs in this population. For instance, the total indirect health-related cost of IBD in Canada in 2018 was estimated to be CAD 1.29 billion. Still, as highlighted by Kuenzig et al., costs related to reduced professional development and personal achievement due to illness remain undetermined [13]. The societal cost of illness of IBD is also increasing due to growing costs of medication and presents geographical differences. For instance, for prevalent CD cases in the last ten years, annual healthcare costs were in Asia USD 4417 whereas in Europe it was USD 12,439 and in North America USD 17,495 [14]. In Canada, prescription drug use accounted for 42% of total direct costs in IBD patients. Moreover, costs of the therapy of IBD continue to rise what is connected with the increased use of biologic agents [15].

Repetitively demonstrated geographical diversity in UC and CD prevalence rates reflect indirectly the role of the lifestyle-attributable factors in IBD pathogenesis. However, the involvement of the environmental causes in the interactions between genetic, immune, and microbiological factors, that eventually lead to gut inflammation, continues to be obscure. Missing evidence on the role of specific environmental factors reduces the capacity of prevention methods and unclear pathogenesis results in challenging diagnostic processes and a lack of effective therapeutic modalities [16]. The recently proposed triple environmental hit concept (priming, modulation, and trigger), described in detail elsewhere, represents a new approach to IBD pathogenesis highlighting the role of environmental triggers and the key role of the microbiome [17].

Furthermore, IBD is distinguished as *systemic disease*. It has to be underlined that clinical manifestation of IBD involves not only classic *extraintestinal manifestations* but also an extended list of *comorbidities*, which are categorised as classic (psoriasis and psoriatic arthritis, psychological and psychiatric disorders, and osteoporosis), emerging (metabolic syndrome and its components, cardiovascular diseases, atherosclerosis, fatigue, chronic obstructive pulmonary disease, sexual dysfunction, and Parkinson’s disease), related to lifestyle (smoking, alcohol consumption, anxiety, and substance misuse), and related to treatment (skin cancer, lymphoma, and dyslipidaemia) [18,19]. As reviewed by Argollo et al. [19], the scale of comorbidities in IBD is unknown, but proper diagnosis of comorbid diseases is crucial as they can interfere with disease activity, impact disease outcome, and influence pharmacological therapy. Moreover, they are related to decreased quality of life, emotional effects, and reduced ability of patients to cope. In addition, IBD is currently more often diagnosed in childhood and adults older than 60 years, which is entirely changing the patients’ profile [20].

One comorbid condition may be a provoking factor for the other one, thus creating the vicious circle. For instance, patients with IBD are exposed to a similar spectrum of the life-style related factors as healthy people. However, IBD negatively influences health-related quality of life and is associated with a higher risk of anxiety and depressive disorders which consequently can lead to increased susceptibility to many kinds of addictions [21]. The strong association between substance use disorders (SUDs), mood, and anxiety disorders was demonstrated in the meta-analysis of 22 unique epidemiological surveys [22]. Yet, in contrast to smoking, the burden of alcohol and narcotics use in IBD is unrevealed and current data referring to addictive substance use is contradictory.

Finally, diet is inseparably connected with IBD. Dietary factors may serve as a link between environment and IBD whereas nutritional interventions are an integral part of therapeutic strategies. Unquestionably, diet plays a significant role in IBD aetiology; however, evidence regarding specific diet components is scanty and indirect [23]. For instance, different incidence rates worldwide, as well as recently observed increase in the IBD occurrence in the regions changing the nutritional habits toward Western types, seem to confirm the significance of diet. Among the proposed mechanisms of action of diet is the influence on the permeability of the mucosal barrier and interactions with components of the immune response. Moreover, diet has an undeniable influence on the gut microbiome and its metabolites, while the microbiome, together with immune and genetic factors, is implicated in the IBD pathogenesis [24,25]

**Figure 1:**
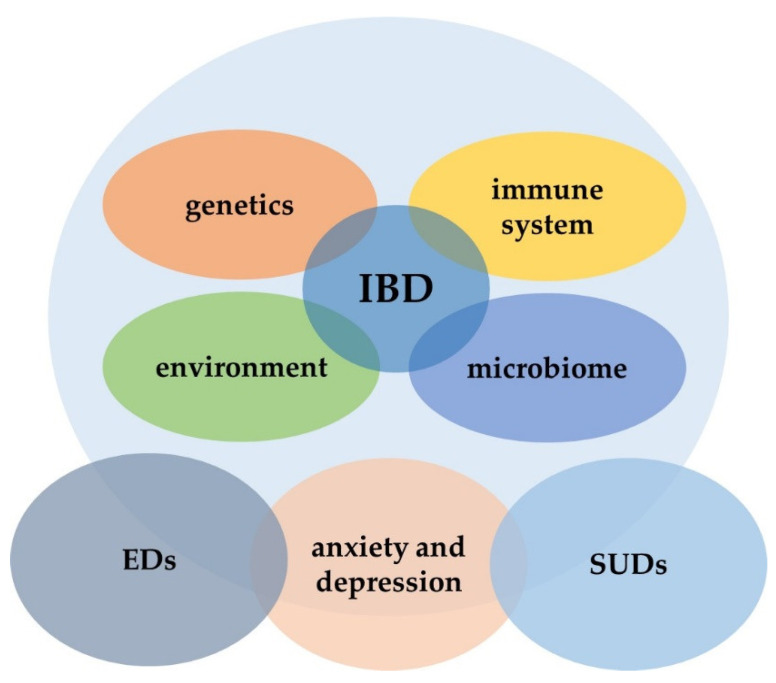
Interactions between IBD and psychiatric disorders. IBD, inflammatory bowel disease; EDs, eating disorders; SUDs, substance use disorders.

Concurrently, interactions between the microbiome, immune system, and nervous system are involved in the pathogenesis of eating disorders (EDs). Alongside some similarities in the pathogenetic ways, the clinical manifestation of EDs may overlap with IBD symptoms. However, little is known about the magnitude of eating disorders in IBD, even if they have the potential to be significant comorbid conditions (Figure 1).

Our review aimed to raise the awareness of comorbidities in IBD patients, which can create a diagnostic challenge and interfere with the therapy and disease outcome.

## 2. Materials and Methods

To review the prevalence of eating disorders, alcohol, and narcotics use disorders in IBD patients, we have searched three publication databases: PubMed, Web of Knowledge, and Embase. We used combinations of the following keywords: (“eating disorders” or “anorexia” or “bulimia” or “alcohol“ or “alcohol use disorders” or “narcotics” or “cannabinoids” or “opioids” or “narcotics use disorders”) AND (“Crohn’s disease” or “ulcerative colitis” or “inflammatory bowel disease” or “IBD”). The search was limited to publications published between January 2010 and June 2021. Duplicate records from the databases were removed before the first eligibility screening. Exclusion criteria were as follows: experimental studies (including animal studies and in vitro research), non-IBD, non-original articles, and non-English language.

## 3. Eating Disorders

### 3.1. Introduction

Eating disorders refer to irregularities in eating behaviour. They are disabling, deadly, and under-researched mental disorders. They typically develop in adolescence with a high preponderance among females [26]. Several distinct forms of maladaptive eating disorders exist, including anorexia nervosa (AN), bulimia nervosa (BN), and binge eating disorders (BEDs) as the most common; however, the list of EDs is still expanding. For instance, avoidant/restrictive food intake disorder (ARFID) has been described recently. Additionally, diagnostic criteria are evolving and currently the *Diagnostic and Statistical Manual of Mental Disorders*—Fifth Edition (DSM-V) is the main investigative device supporting EDs diagnosis. According to the DSM-V criteria, ARFID refers to patients who restrict their dietary intake due to concerns regarding the aversive consequences of eating. Further, ARFID must interfere with nutrition or sufficient energy intake and must occur in the absence of any weight or shape concerns or distorted body image [27].

### 3.2. EDs and IBD—What Do They Have in Common?

EDs and IBD predominantly affect young people [6,26]. Both conditions may manifest with similar signs—weight loss and gastrointestinal symptoms. Furthermore, basic laboratory tests can reveal similar abnormalities resulting from malnutrition. This can create difficulties not only in the differential diagnosis but mainly in the establishment of coexistence. Malnutrition is common in AN and IBD, especially in Crohn’s disease patients. As reviewed elsewhere, malnutrition is affecting up to 70% of patients with active IBD and up to 38% of patients in remission. Moreover, it is linked with increased hospitalisations, disease flares, need for surgery, and post-operative complications. Early identification of malnutrition by applying nutritional screening tools is pivotal [28]. Surveillance of nutritional status in IBD patients is mandatory regardless of age [29]. Malnutrition in IBD has a complicated background, yet the inadequate dietary intake, usually secondary to avoidance of eating during the active phase of the disease to control symptoms or exclusion of certain food, plays an essential role [30].

Further, microbiome alterations and inflammation are fundamental features of IBD. However, microbiome perturbations and dysregulation of the immune system were also demonstrated in EDs patients. Nevertheless, microbiome changes are linked to a wide range of diseases [31], and the evidence is missing if the dysbiosis in EDs and IBD share similar patterns. Therefore, according to the current knowledge, only the fact of the involvement of microbiome in both groups of diseases is common, but not specific microbiome alterations.

Anorexia nervosa is linked with proinflammatory dysregulation, whereas bulimia nervosa is associated with a decreased level of proinflammatory cytokines. Observed alterations can be only partly explained by the nutritional status, namely undernutrition and obesity. Therefore, the microbiome was proposed to be the key player in this complex process; however, further research on the pattern of dysbiosis and its role in EDs is desirable [32].

For instance, deep alterations of microbiota in anorexia nervosa, represented by higher levels of mucin-degraders and members of Clostridium clusters I, XI, and XVIII and reduced levels of the butyrate-producing *Roseburia* spp. were demonstrated. Interestingly, microbial perturbations in patients with restrictive and binge/purging AN-subtypes were distinct. Changes in microbiota composition were complemented with increased concentrations of branched-chain fatty acids (BCFA), which are indicators for protein fermentation. Microbiota alterations did not recover after weight gain and/or normalisation of eating behaviour. Furthermore, the authors confirmed that gastrointestinal symptoms are common in AN patients; however, they also noted that the majority of them did not improve after weight gain [33].

Conversely, eating behaviour in gut inflammation results from the complex interaction between gut peptides, changed gut–brain communication, cognitive and psychological factors, disease symptoms, and inflammation [34]. The issue of the diet is far beyond the pathogenesis and therapy. It influences the disease course, nutritional status, and quality of life. For instance, it was shown that higher disease activity and avoiding certain foods during disease flares were linked with a high risk of malnutrition [35].

Finally, there is an association between eating disorders and psychiatric comorbidities. For instance, a strong association of EDs with other psychiatric comorbidities was shown in a cross-sectional survey of a nationally representative sample of over 10,000 adolescents aged 13 to 18 years [36]. Concurrently, as psychiatric disorders are prevalent in IBD patients it is another argument for further research on the coexistence of these disorders.

### 3.3. ED and IBD—Diagnostic Challenge

**Figure 2:**
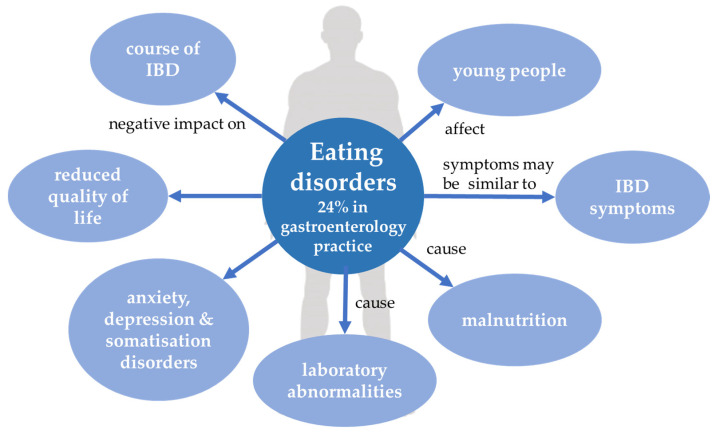
Eating disorders and inflammatory bowel disease. IBD, inflammatory bowel disease.

As summarised by Werlang et al. several reasons are making EDs important for gastroenterologists: common prevalence (10% in general population and even 24% in gastroenterology practice), association with reduced quality of life, development of anxiety, depression, and somatisation disorders as well as a number of complications which may also involve the gastrointestinal tract [37]. Furthermore, recommended laboratory tests in adolescence and young adults with weight loss and suspicion for anorexia nervosa include complete blood count, ESR, serum chemistry panel, liver function tests, albumin, thyroid function tests (thyroid stimulating hormone, free thyroxine, and triiodothyronine), urinalysis, and 25-hydroxyvitamin D [38]. Nevertheless, besides the expected obvious differences in the results of the above-mentioned tests between anorexia nervosa and IBD patients, resulting mainly from the presence of inflammation in IBD, they are not effective in making the differential diagnosis. First of all, for instance, anaemia and decreased concentration of albumin may be present in both conditions. Secondly, there is possible coexistence of both EDs and IBD. It has to be highlighted, that there are at least two clinical scenarios in adolescence and young adults with gastrointestinal symptoms and weight loss. Eating disorders may mimic gastrointestinal disease, mainly IBD and celiac disease, but EDs may also coexist with gastrointestinal disease. Thus, diagnostic workup should be extended to include more than the laboratory tests; a multidisciplinary approach to the patients is essential for proper diagnosis. Establishing a dual diagnosis is also challenging because of the overlapping of clinical symptoms, mainly weight loss. Furthermore, in patients with IBD diagnosis ongoing weight loss despite optimal control of disease activity should be an alarming symptom and raise suspicion towards EDs, especially among patients reporting satisfaction with current weight, normal dietary intake, and good medical compliance [39]. Interrelations between EDs and IBD are demonstrated in Figure 2.

Therefore, recognising the scale of the problem of EDs in the population of IBD patients is reasonable. However, there is little available data, especially regarding ARFID. Literature on EDs in IBD is dominated by case reports and case series presenting diagnostic difficulties or coexistence of both conditions. In a systematized review published in 2017, the most frequent combination reported in the literature was anorexia nervosa and Crohn’s disease, which accounted for 90 cases. Comorbid EDs and IBD, as expected, affected mostly young females. Another finding which can be important in clinical settings was that IBD was used to potentiate weight loss [40].

### 3.4. EDs in IBD Patients

In the light of the trend of increased incidence of IBD in children the findings from the recently published study of Butwicka et al. sounds alarming. In total, 17.3% of almost 6500 patients with childhood-onset IBD during the median follow-up time of 9 years received a diagnosis of a psychiatric disorder, compared to almost 12% in the general population. Furthermore, results were confirmed by sibling comparison. Eating disorders (HR, 1.6; 95% CI, 1.3–2.0) were reported next to the other psychiatric problems, including suicide attempt (HR, 1.4; 95% CI, 1.2–1.7), mood disorders (HR, 1.6; 95% CI, 1.4–1.7), anxiety disorders (HR, 1.9; 95% CI, 1.7–2.0), personality disorders (HR, 1.4; 95% CI, 1.1–1.8), attention-deficit/hyperactivity disorder (HR, 1.2; 95% CI, 1.1–1.4), and autism spectrum disorders (HR, 1.4; 95% CI, 1.1–1.7). Moreover, the hazard ratios for the psychiatric disorders were highest in the first year of follow-up. The other very important findings from the clinical point of view are connected with the identification of the factors associated with the increased risk of psychiatric disorder in IBD patients. There are as follows: very early onset IBD (<6 years of age); parental psychiatric history as well as factors corresponding with more severe disease course—extraintestinal manifestations; bowel surgery; and perianal surgery. A number of outcomes for eating disorders in the IBD group and the general population was 1.1 and 0.7 per 1000 person-years, respectively. Furthermore, when compared with siblings without IBD, the frequency was 1.3 and 0.7 per 1000 person-years, respectively. Unfortunately, the authors did not include the prevalence of specific EDs, so data, however exceptional and valuable, still do not shed the light on the details of EDs in IBD patients. A study delivered significant arguments for an interdisciplinary approach and psychiatric screening in IBD patients [41].

Results of the study presented in the form of an abstract demonstrated that 24.3% of 95 IBD patients met the criteria for an eating disorder: anorexia nervosa, bulimia nervosa, or binge eating disorder based on the questionnaire Eating Disorders Diagnostic Scale. This rate was higher when compared to prevalence on the level 2.7% in the general population. Authors observed that the patients with positive screening had a higher BMI, which may be responsible for a more challenging diagnosis. Data were presented as an abstract, which limits the interpretation [42]. In another study, 16% of 83 patients had scores above the clinical threshold in the screening diagnostic tool Eating Attitude Test-26. However, the results were again presented as an abstract [43].

Studies repeatedly displayed a high prevalence of psychiatric disorders in IBD patients. Still, it is a striking finding that often they remained undiagnosed. A recently published study demonstrated not only that almost 50% of IBD patients had at least one psychiatric disorder, but also that in almost 60% of patients the diagnosis was made for the first time during participation in the study. Authors confirmed that the most common disorders refer to mood disorders (47%) and anxiety (24%); however, they also found out that 5 patients from 237 (4/136 CD) met the criteria for bulimia [44]. In the small series of IBD cases (*n* = 10) authors demonstrated the overlap of ARFID and IBD and persistence of EDs symptoms despite effective IBD therapy. Interestingly, EDs symptoms developed in the majority of patients after the IBD diagnosis [45] (Table 1).

### 3.5. IBD in EDs Patients

Studies focused on the non-psychiatric comorbidities in patients with eating disorders or only in patients with anorexia nervosa, shed the light on the occurrence of inflammatory bowel disease IBD in patients with disordered eating. For instance, a 21-year follow-up study on comorbidities in a group of 169 female inpatients with anorexia nervosa revealed three cases of IBD. Authors analysed AN outcomes depending on the presence of comorbidities, which were then further classified as related to AN and independent of AN. Significantly, mortality was more common among inpatients with somatic comorbidity [46]. Results of the study delivered argumentation for careful evaluation of the EDs patients toward comorbidities as they might negatively impact the outcome of the disease. Likewise, results of the large Finnish cohort study (2342 patients with EDs) demonstrated a higher risk of autoimmune diseases in patients with EDs. Remarkably, the risk of gastroenterological diseases was attributed mainly to Crohn’s disease (OR 1.8, 95% CI 1.4–2.5, *p* < 0.001). Crohn’s disease was diagnosed in 1.1% of EDs patients and 0.4% of control individuals (*p* < 0.001). Ulcerative colitis was diagnosed in 1% of EDs and 0.7% of the control group (*p* = 0.16). As the study was focused on multiple autoimmune diseases, the observed coexistence of a wide number of autoimmune disorders with EDs is raising the possibility of a common background of the diseases [47]. Correspondingly, a retrospective, record-linkage cohort study in England revealed a strong association between EDs and specific autoimmune diseases. Females admitted to hospital with AN had a two-fold elevation of risk of hospitalization with a subsequent autoimmune disease (RR 2.0, 95% confidence interval 1.8–2.3) whereas females admitted with the autoimmune disease had a three-fold elevation risk of hospitalization with AN. Females with AN had elevated risks of Crohn’s disease and ulcerative colitis. The study was conducted in a large cohort study; however, it was limited to hospitalized patients, which may influence the obtained results [48] Table 2).

## 4. Alcohol

According to the World Health Organisation (WHO) [49], the harmful use of alcohol is one of the prominent risk factors for population health globally. There is a diversity in the total alcohol per capita consumption worldwide and the highest levels are currently reported in the WHO European Region and expected to increase in the Americas, South-East Asia, and the Western Pacific. Nevertheless, total alcohol per capita consumption globally among people over 15 years of age was at the level of 6.4 litres of pure alcohol in 2016. The other remarkable observation demonstrated that 26.5% of all 15–19-year-olds are current drinkers, which in light of the findings that drinking of alcohol in childhood and adolescence is associated with a higher risk of alcohol use disorders in adulthood is alarming [50]. The scale of the problem is further reflected by 3 million deaths worldwide in 2016 resulting from the harmful use of alcohol. More than 20% of all deaths attributable to alcohol consumption worldwide were due to digestive diseases and almost 13% due to cancers [49]. Alcohol is a group 1 carcinogen and there is evidence that alcohol is a cause of cancers of the oral cavity, pharynx, larynx, oesophagus, colorectum, liver, and breast [51]. Multidirectional strategies in answer to this crisis include many public efforts and recommendations for screening excessive alcohol consumption to identify individuals with the increased risk of alcohol-attributable harms [52].

### 4.1. Alcohol and IBD

The potential interplay between alcohol and IBD may be present on several levels: disease pathogenesis, disease course, disease complications such as malignancies, and malnutrition.

#### 4.1.1. Alcohol and Pathogenesis of IBD

Alcohol is a factor considered to be an immune modulator. It affects intestinal permeability leading to leakiness and intensified transfer of microbiota from the gut to the blood [53]. This type of qualitative and quantitative alterations in gut bacteria play a crucial role in IBD pathogenesis but also can destabilise the function of other organs [54,55]. Among individuals who use alcohol chronically, increased permeability can promote transient endotoxemia [56]. Furthermore, previous experimental studies conducted on individuals who consumed alcohol chronically revealed that in these cases Kupffer cells and leukocytes activity can be increased leading to hyperproduction of proinflammatory factors such as tumour necrosis factor-alpha (TNF-alpha), interleukin 1 (IL-1), and interleukin 6 (IL-6) [57,58].

Interestingly, some kinds of alcoholic beverages such as red wine—due to its anti-inflammatory and antioxidant properties—can reduce inflammation, which is reflected in reduced faecal calprotectin levels [59]. Red wine alters gut microbiota leading to an increase of anti-inflammatory bacterial groups such as *Bifidobacterium*. It was not proven in the case of other alcoholic beverages (e.g., gin) that tend to increase proinflammatory species such as *Bacteroides* and *Clostridium* [59]. A case–control study of 354 individuals conducted in China showed a protective effect of light alcoholic beverages consumption against the development of UC [60]. However, due to the obvious link between alcohol and chronic heart or liver disorders, according to the current guidelines for dietary management in IBD, alcohol, generally, should be avoided [61].

Some studies are supporting the role of alcohol consumption in the pathogenesis of IBD. For instance, one of the largest matched-cohort studies in Taiwan with over 250,000 participants showed that individuals hospitalized for alcohol abuse had a higher risk of developing IBD in the next 10 years compared to the control group [62]. Nevertheless, this observation has not been confirmed in the newest, recent studies. According to them, there is no evidence that alcohol consumption may increase the risk of IBD development. In the European questionnaire-based study the relationship between alcohol consumption and the development of IBD was not found after 4 to 10 years of follow-up [63]. Further, a meta-analysis of nine studies on beverage consumption and risk of UC did not reveal an association between alcohol and risk of developing UC (OR, 0.95; 95% CI, 0.65–1.39) [64]. Similarly, a meta-analysis of six studies in CD also found no association between alcohol and risk of CD [65].

#### 4.1.2. Alcohol and IBD Outcome

Although last studies suggest that evident correlation does not exist, the connection between alcohol intake and IBD course is significant. Alcohol can be classified as a modifiable risk factor of relapse [63,66,67]. As previously mentioned, its consumption may contribute to the increased concentration of proinflammatory mediators leading to the flare by the formation of crypt microabscesses, mucosal ulceration, and damage of colonic epithelium [68,69]. The risk of flare is distinctly higher in the case of sulphite-rich beverages intake like wine and beer that particularly increase epithelial permeability and cause histologic changes in animal models’ colons similar to changes seen in humans with UC [56,70,71]. In addition, it must be underlined that alcohol as an irritating substance may cause many gastrointestinal symptoms including reflux and abdominal pain that potentially can be misleading in the diagnosis and treatment of IBD [72].

Alcohol intake has been negatively linked with the outcome of hospitalized patients with IBD increasing the number of computer tomography scans of the abdomen, the number of intestinal biopsies obtained, the frequency of intestinal infections, and the need for antibiotics injections [56].

A number of studies demonstrated that alcohol consumption is a factor associated with worsening of symptoms in IBD patients [73]. For instance, the US survey study showed that 40% of participants reported worsening of symptoms following alcohol intake [74]. Similar research from New Zealand revealed that 55% of patients with CD complained of exacerbation of symptoms after beer consumption. Similarly, in the recent study of Guida et al., almost 19% of patients perceived alcohol as a symptoms trigger [75].

Selected studies on the impact of alcohol consumption on IBD outcome are presented in Table 3.

Alcohol is among the most often avoided dietary factors reported by IBD patients. For instance, in the prospective study on dietary practices and beliefs in older-onset IBD patients alcohol together with spicy and fatty foods, carbonated drinks, red meat, and raw fruit and vegetables were reported as the most common avoided dietary component [81]. Similar findings were reported in studies on another group of IBD patients, such as 255 British South Asians with IBD or 205 patients with inactive UC [82,83]. These findings are in the line with the previous research on dietary practices and beliefs in IBD patients. Limdi et al. demonstrated not only that alcohol was avoided by 22% of IBD patients but also it was implicated by 21% of patients in worsening the symptoms [80]. In the University of Manitoba’s IBD Cohort Study, alcohol, popcorn, legumes, nuts, seeds, deep-fried food, and processed deli meat avoidance was common in IBD patients, especially in individuals with active disease [84]. Alcohol avoidance is usually linked with its negative impact on symptoms.

#### 4.1.3. Alcohol and Complications of IBD

Chronic alcohol drinking has a strong impact on transit time in the gut leading to diarrhoea and consequently malnutrition. Malnutrition is commonly observed among alcohol users and is additionally caused by impaired absorption of many ingredients such as thiamine, folate, and vitamins [85].

Furthermore, alcohol alters the hepatic metabolism of many drugs influencing the ethanol oxidizing system, particularly cytochrome P450. This is an important aspect concerning the chronic treatment of IBD [86]. Many of IBD-specific drugs are described to interact with alcohol [87]. The most important clinical implications of simultaneous use of ethanol with medications are summarized in Table 4.

#### 4.1.4. Alcohol Consumption in IBD Patients

Current data relating to alcohol consumption in the IBD population is still insufficient and further investigation is needed to assess the real scale of the problem. The majority of studies are focused on psychiatric disorders in IBD patients and data on alcohol use disorders are general as authors do not analyse specific patterns of alcohol consumption and register the presence of substance or alcohol use disorders.

Increased risk for SUDs was demonstrated in studies in paediatric and young adults with IBD [95]. It is worth noting that in another study in adolescents and young adults with IBD majority of them (76/132) were abstainers; however, those reporting usages of marihuana and binge drinking episodes have been also smoking cigarettes [96]. Furthermore, two large studies from Sweden on early onset IBD and adult-onset IBD shown a higher prevalence of substance misuse compared to both population and sibling controls [41,97]. In turn, a Canadian study sheds light on the issue of higher risk of alcohol misuse in women in the postpartum period [98].

According to previous studies on a US population of 129 individuals with IBD, the quantity and pattern of alcohol consumption are similar as in the general population. Taking into account the criteria of the National Institute of Alcohol Abuse and Alcoholism almost half of patients were abstainers (46% of CD and 42% of UC) [76]. Nevertheless, referring to one of the Canadian studies, alcohol is a substance that is most often used and causing dependence among IBD patients (Table 5).

**Figure 3:**
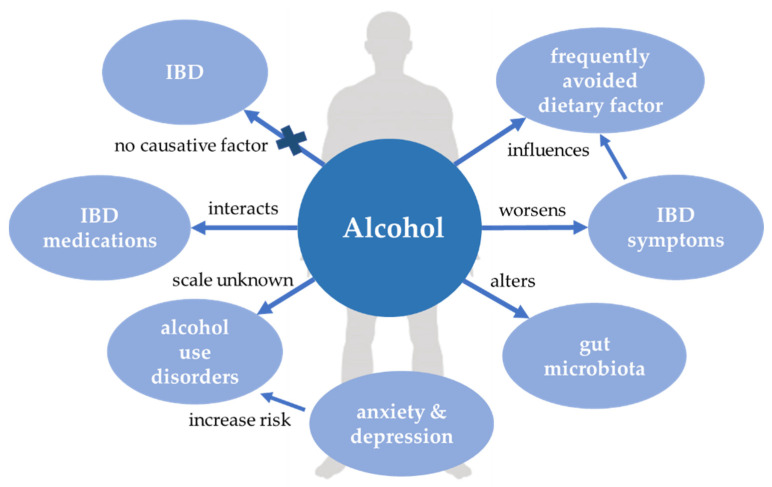
Alcohol and inflammatory bowel disease. IBD, inflammatory bowel disease.

Interrelations between alcohol and IBD are presented in Figure 3.

## 5. Narcotics

### 5.1. Narcotics Use in IBD Patients

Comparing to other chronic conditions, IBD patients are more inclined to health risk behaviours (HRBs) [99]. A Canadian study on a group of 247 participants showed that one in six persons with IBD experienced a substance use disorder (SUD). Drug dependence was identified in 3.6% of studied IBD patients. Substance use was reported by 52 patients (21.1%): cannabis 48 (19.4%), stimulants 4 (1.6%), opioids 4 (1.6%), cocaine 7 (2.8%), and hallucinogens/PCP 9 (3.6%). Factors contributing to SUD were smoking, lifetime anxiety disorder, higher pain impact, and male sex. Authors suggest that narcotic use can be an indicator of more severe disease because it is associated with increased disease activity and decreased HRcQoL [100]. Another study among individuals with Crohn’s disease assessed that the greatest probability of narcotic intake was among females with higher rates of disability, a longer duration of disease, and a history of many previous surgeries [101]. There are few sources evaluating narcotics use (other than cannabinoids) on the IBD course.

### 5.2. Opioids

Pain often accompanies IBD patients. In one of the Swiss studies, more than 70% of individuals have experienced pain during the disease course reporting mainly back and abdominal complaints. A significant part of them suffered from a long-standing problem (>5 years) [102]. Chronic pain implies the need for analgesic treatment, including opioids. These drugs are widely prescribed to IBD patients worldwide. In one six-years-long study almost half of the participants have received a prescription for an opioid analgesic [103]. Frequent occurrence of pain explains why IBD is an independent risk factor for opioid misuse. Consequently, chronic use of opioids among a significant number of representatives from this group is associated with adverse outcomes, including premature mortality [104]. Individuals classified as heavy opioids users are exposed to many side effects for instance nausea, constipation, cardiovascular incidents, intestine obstruction, or narcotic bowel syndrome.

The opioid system is composed of u-, kappa-, and delta-opioid receptors and their endogenous ligands (met-enkephalin, leu-enkephalin, β-endorphin, and dynorphin) which influence GI functions and, according to previous studies, play an important role in the intestinal inflammation process [105].

Although in IBD therapy opioid agonists are the most often used, there are some studies confirming the efficacy of opioids inhibitors. Low dose naltrexone used in patients with recurrent or refractory IBD induced clinical improvement or remission in a significant number of individuals participating in one of the Dutch studies. The mechanism of action of the drug is based on the amelioration of epithelial barrier function by improving wound healing. Since the low-dose naltrexone is safe and seems to be effective in problematic IBD cases further investigations are required to consider this drug as an option in standard therapy. Some sources even suggest that low dose naltrexone is a big hope to limit the use of anti-inflammatory and immunosuppressive drugs [106,107].

Considering the side effects of opioids and the risk of misuse, leading gastroenterology associations to advise avoiding using them for a long time in cases of IBD-related abdominal pain [108].

### 5.3. Cannabinoids

The interest in cannabinoids use as a way of IBD treatment has become higher recently [109]. Cannabinoids are involved in the regulation of gastrointestinal motility, secretion, and immune tolerance. They act through the body’s endocannabinoid system (ECS) consisting of cannabinoid 1 (CB1) and 2 (CB2) receptors and their endogenous ligands (endocannabinoids) such as arachidonoylglycerol (2-AG), anandamide (AEA), or ligands’ synthesizing and degrading enzymes. Moreover, there exist differences from CB1/CB2 receptor’s sensitive to endocannabinoids which modulate CB receptors’ function. The whole system is called the “endocannabinoidome” and all its elements can be found in the gastrointestinal tract. Furthermore, some components are located in the brain which is significant in the central regulation of gut functioning and confirms possible manipulation of the brain–gut axis by cannabinoids intake. For example, intracerebroventricular activation of CB receptors in mice leads to inhibition of intestinal transit. According to another study genetic deletion of one of the CB ligands significantly modified the composition of the gut microbiota [110,111,112].

There were clinical trials with using of medical marijuana in IBD patients that showed mainly subjective improvement reflecting appetite and sleep. Another study also confirmed only subjective feelings and showed that 63% of participants reported an improvement of symptoms and 67% an improvement in quality of life. Meanwhile, 81% of them would recommend cannabis use for IBD [113]. Although clinical response in CD and UC has been described many times, recent studies have not confirmed cannabinoid influence on objective outcomes like mucosal healing or concentration of inflammatory factors [114,115].

Since cannabinoids are well known as a factor causing relief in IBD, patients tend to try self-treatment. This phenomenon is significant because they rarely inform physicians about alternative ways of therapy. The frequency of cannabinoid use is shown in Table 6.

Patients with IBD self-treated with cannabis tend to have increased vulnerability to substance misuse when compared with those using cannabis recreationally. This dependence or overusing reflects in the higher levels of impulsivity measured with the Substance Use Risk Profiles Scale (SURPS), depressive symptoms, and stronger motivation to use cannabis for coping [124]. Moreover, according to the previous studies, IBD patients have been reported to have a higher prevalence of depression and anxiety among cannabis users [118]. It is also suggested that there exists a correlation between cannabis use and illicit substances such as cocaine. Ten percent of past or current cannabis users reported to use illicit drugs within the past 6 months, compared with 0% of individuals who never used cannabis [125]. Some studies report that marijuana use is associated with a worse prognosis in patients with CD, meanwhile, it can reduce symptoms of UC [126]. Another study does not confirm any connection between advancement of the inflammation process assessed by biomarkers level among cannabis users [127].

The poor outcome, potential risk of psychiatric, mood, and cognitive disorders exclude the use of cannabinoids as a regular, recommended therapy [114]. In addition, the dose of potentially beneficial cannabinoid therapy is also discussed. The last study has shown no effect of low-dose treatment. It suggests the necessity of using higher doses but they cannot be accepted for medical treatment since previous studies have been conducted on patients using cannabinoids by smoking [115,128].

Possibly in the future, other endocannabinoids ligands that do not affect the central nervous system will be an option for IBD patients.

**Figure 4:**
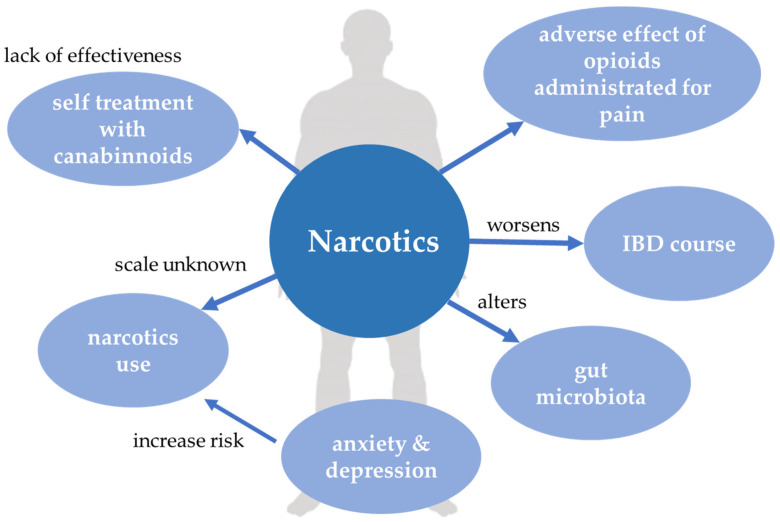
Narcotics and inflammatory bowel disease. IBD, inflammatory bowel disease.

Interrelations between narcotics and IBD are displayed in Figure 4.

## 6. Psychiatric Disorders and SUDs

Importantly, susceptibility to narcotic misuse is often associated with personality or psychiatric disorders. For instance, one study showed that 38% of drug-dependent patients were also diagnosed with borderline personality [129]. Many individuals had a psychiatric diagnosis before the IBD diagnosis [130]. Since the prevalence of anxiety and depression in IBD is higher than in the general population, it can be expected that this population is more exposed to psychoactive substances. Even among child patients, 5.6% of IBD individuals were reported as chronic narcotics users, while the frequency of narcotics use was strongly correlated with comorbid mental health problems [131].

## 7. Conclusions

Eating disorders might be added to the list of comorbidities of IBD. First, both conditions share a complex background with the special role of an environment and the gut microbiome. Secondly, IBD is related to an increased risk of psychiatric comorbidities, mainly mood disorders and anxiety, which may, in turn, predispose one to the development of EDs. However, a lesson learned from research on psychiatric disorders in IBD is that they are still underdiagnosed. The prevalence of EDs and IBD is rising worldwide [132,133,134,135]. This most probably reflects environmental changes. Finally, it has to be highlighted that overlapping clinical symptoms and laboratory test abnormalities can cause difficulties in the recognising coexistence of both conditions.

Alcohol use among IBD patients is a not fully elucidated issue. It is one of the most frequently avoided dietary factors and it is connected with the worsening of symptoms. However, IBD patients, due to the higher incidence of psychiatric disorders, are at higher risk to develop alcohol abuse disorders.

Similarly, the global problem of narcotic use in IBD individuals remains uncharted. In the last few years, researchers have tried to find a positive impact of cannabinoids on the IBD course. Although subjective feelings of patients are satisfying, no evidence confirms without any doubts any beneficial impact on the underlying disease. Considering the side effects of opioids and the risk of misuse, leading gastroenterology associations advise avoiding using them for a long time in cases of IBD-related abdominal pain.

Further studies on eating disorders and substance use disorders in IBD patients are needed. Research on these conditions together with other psychiatric comorbidities, using validated diagnostic tools and in the strict cooperation between gastroenterologists and psychiatrists could shed a new light on this issue and help to identify the patients at high risks and may benefit from early diagnosis and therapy.

## Figures and Tables

**Table 1:** Eating disorders in inflammatory bowel disease patients.

Study	Author	IBD Patients	EDs Patients *n* (%)	Tool	Remarks
Prospective cohort study [44]	Marafini et al. 2020	101 UC and 136 CD	5 (4.9) BN 4 (2.9) BN/136 CD	MINI 5.0.0 *	single university hospital; Italy
Retrospective cohort study [41]	Butwicka et al. 2020	6464	83 (1.3)	ICD codes	early onset IBD; compared to general population and siblings without IBD; Sweden
Cross-sectional study [43]	Wabich et al. 2020	83	13 (15.7) patients scoring above threshold when clinical evaluation is recommended	survey with EAT-26	abstract
Cross-sectional study [42]	Robelin et al. 2020	95	23 (24.2)	survey with EDDS	academic outpatient IBD practice; abstract; United States

EDs, eating disorders; IBD, inflammatory bowel disease; EDDS, Eating Disorders Diagnostic Scale; EAT-26, Eating Attitude Test-26; BN, bulimia nervosa; CD, Crohn’s disease; UC, ulcerative colitis; ICD, International Classification of Diseases; MINI 5.0.0, Mini International Neuropsychiatric Interview 5.0.0. * The MINI 5.0.0 provides psychiatric diagnoses based on the *Diagnostic and Statistical Manual of Mental Disorders* IV Text revision (DSM-IV-TR) criteria.

**Table 2:** Inflammatory bowel disease in eating disorders patients.

Study	Author	IBD	EDs	Patients with EDs and IBD (%)	Remarks
Retrospective cohort study [46]	Erdur et al. 2012	3	169	1.8	limited to females with AN; 21 years follow-up; Germany
Retrospective cohort study [47]	Raevuori et al. 2014	27 CD/2315 24 UC/2318	2342	UC: 1 CD: 1.1	patients from special care centre; compared to the general population; Finland
Retrospective cohort study [48]	Wotton et al. 2016	42 CD and 36 UC in AN females as the first admission 41 CD and 26 UC in AID as the first admission 15 CD and 7 UC in BN as first BN admission 17 CD and 15 UC in AID as first admission	8700 females and 651 males with AN 4783 females and 330 males with BN	AN: 0.83 BN: 0.43	a study using national administrative statistical data on hospital care and mortality, 1999–2011; limited to hospitalized patients; United Kingdom

IBD, inflammatory bowel disease; EDs, eating disorders; AN, anorexia nervosa; BN, bulimia nervosa; CD, Crohn’s disease; UC, ulcerative colitis; AID, autoimmune disease.

**Table 3:** Impact of alcohol on inflammatory bowel disease outcome.

Study	Author	IBD Patients	Method	Major Findings
Cross-sectional study	Jowett et al. 2004	183 inactive UC	Food frequency questionnaire Simple clinical colitis activity index	High intake of alcohol as well as high intake of sulfur and sulfate were associated with increased risk of UC relapse.
Cross-sectional study	Swanson et al. 2010 [76]	52 inactive CD 38 inactive UC 39 IBS	Validated questionnaire of National Institute of Alcohol Abuse and Alcoholism Novel questionnaire evaluating the effect of alcohol on symptoms	Worsening of symptoms with alcohol in current drinkers No correlation between the type of alcoholic beverage and symptoms.
Cross-sectional study	Triggs et al. 2010 [77]	446 CD	An extensive dietary questionnaire (257 food items in 15 groups) recorded self-reported dietary tolerances and intolerances	Beer and wine had adverse effects in more than 45% of CD patients.
Cross-sectional study	Zallot C. et al. 2012 [78]	244 IBD	Questionnaire of 14 items	Only 6 patients reported alcohol as avoided factor to prevent relapse.
Cross-sectional study	Cohen et al. 2013 [79]	1121 CD 405 CD ostomy patients 597 UC 206 UC pouch patients	Semi-quantitative food frequency questionnaire to measure dietary consumption patterns and open-ended questions to elicit responses from patients about food items they believe ameliorate or exacerbate IBD	Alcohol made symptoms worse in CD, UC, and UC patients with pouch.
Cross-sectional study	Limdi et al. 2016 [80]	400 IBD patients	A questionnaire assessing demographics, dietary beliefs, and habits in IBD patients	Alcohol was implicated in the worsening of symptoms in 21% of patients.
Cross-sectional study	Guida et al. 2021 [75]	81 CD 86 UC most patients were in remission or had mild disease activity	A semi-structured questionnaire consisting of 48 questions	18.6% of patients perceived alcoholic drinks as a symptoms trigger.

IBD, inflammatory bowel disease; UC, ulcerative colitis; Crohn’s disease; CD; IBS, irritable bowel syndrome.

**Table 4:** Interactions of IBD-specific medications with alcohol.

Group of Medications	The Chemical Name of the Active Substance	Mechanism of Interaction	Possible Clinical Implications
Antibiotics	Metronidazole	Disulfiram-like—inhibition of aldehyde dehydrogenase [88]	Facial flushing, diaphoresis, tachycardia, hypotension, abdominal pain, pounding headache
Immunomodulator/immunosuppressants	5-aminosalicylates	Interference with modified-release formulations [89]	Reduced effectiveness
	Cyclosporine	Decreased or increased drug-circulating level [90]	Reduced effectiveness or toxicity
	Azathioprine	Glutathione depletion in hepatic endothelial cells leading to increased azathioprine toxicity [91]	Peliosis hepatis
	Methotrexate	Direct hepatotoxicity via inhibition of DNA and RNA synthesis [92,93]	Liver injury
Analgesics	Paracetamol	Increased metabolism of paracetamol by CYP2E1 [94]	Higher risk of hepatotoxicity

DNA, deoxyribonucleic acid; RNA, ribonucleic acid; CYP2E1, cytochrome 2E1.

**Table 5:** Alcohol misuse in inflammatory bowel disease patients.

Study	Author Year	IBD Patients	Major Findings
Cross-sectional study	Swanson et al. 2010 [76]	129 patients with inactive disease (52 CD and 38 UC)	Abstainers: 38% CD; 37% UC Current drinkers 62% CD; 63% UC Light drinkers: 21% CD; 26% UC Moderate drinkers: 33% CD; 24% UC; Heavy drinkers: 0% CD; 3% UC; Binge drinkers: 19% CD;16% UC
Cross-sectional study	Plevinsky et al. 2019 [96]	132 adolescents and young adults (age 16–25 years)	Substance use (tobacco use, marijuana use, and binge drinking) in the last 30 days: global users (*n* = 17), marijuana users engaging in binge drinking (*n* = 18), exclusive binge drinkers (*n* = 21), and global abstainers (*n* = 76) Older age, male gender, active disease, at least 1 hospitalization in the past year, low self-efficacy, low HRQoL, and high adherence barriers were significantly more likely for those reporting multi substance use. All those reporting both marijuana use and binge drinking also reported tobacco use.
Case control study	Thavamani et al. 2019 [95]	IBD (*n* = 58,020) Controls (*n* = 11,258,430) age 5–24 years	Prevalence of psychiatric disorders 21.6% The odds ratio for substance misuse * 2.78
Retrospective cohort study (Canada)	Vigod et al. 2019 [98]	New-onset mental illness from conception to 1-year postpartum was compared between 3721 women with and 798,908 without IBD	Women with IBD were at an increased risk of new-onset psychiatric diagnosis in the postpartum period, but not during pregnancy. The risk was specifically elevated for a new-onset mood or anxiety disorder (aHR 1.14, 95% CI 1.04 to 1.26) and alcohol or substance use disorders (aHR 2.73, 95% CI 1.42 to 5.26).
Retrospective cohort study	Butwicka et al. 2020 [41]	6464 early onset IBD patients	Frequency and Absolute Incidence Rates per 1000 Person-Years (Incidence Rate): IBD patients: 250/70,343 (3.6) compared to population: 11,682/3,531,789 (3.3); IBD patients: 175/49,178 (3.6) compared to siblings: 187/72,500 (2.6)
Retrospective cohort study (Sweden; 1973–2013)	Ludwigsson et al. 2021 [97]	69,865 adult-onset IBD patients (UC: 43,557; CD: *n* = 21,245; IBD-unclassified: *n* = 5063) compared to 3,472,913 general population references and 66,292 siblings	Absolute incidence rates per 1000 person-years for substance misuse * in patients with adult-onset IBD (97,232/47,852,723) compared with matched reference individuals (2065/925,841; *p* = 0.0002 and IBD patients (1202/528,668) compared with their siblings without IBD (1913/964,487; <0.0001).
Cross-sectional study	Carney et al. 2021 [99]	247 IBD	SUD ** 16.6% Factors associated with elevated odds of SUD were ever smoking (adjusted odds ratio [aOR], 2.96; 95% confidence interval [CI], 1.17–7.50), male sex (aOR, 2.44; 95% CI, 1.11–5.36), lifetime anxiety disorder (aOR, 2.41; 95% CI, 1.08–5.37), and higher pain impact (aOR, 1.08; 95% CI, 1.01–1.16). Alcohol abuse was the most common lifetime SUD diagnosis (9.3%), followed by alcohol dependence (7.3%) and drug abuse (7.3%).

IBD, inflammatory bowel disease; CU, Crohn’s disease; UC, ulcerative colitis; SUD, substance use disorders; HRQoL, health-related quality of life; * not specified type of the substance. ** Substance use disorders were defined by meeting diagnostic criteria for current or lifetime SUD and categorized by DSM-IV diagnoses: alcohol abuse, alcohol dependence, drug abuse, and drug dependence.

**Table 6:** The frequency of cannabinoids uses among inflammatory bowel disease patients.

Study	Author, Year	IBD Patients	Method	The Frequency of Cannabinoids Use (%)
Cross-sectional study	García-Planella et al. 2007 [116]	214	Anonymous, structured questionnaire, administered to consecutive patients with IBD of at least 2 years of duration, seen in an IBD outpatient clinic	10
Cross-sectional study	Lal et al. 2011 [117]	UC: 100 CD: 191	Questionnaire regarding current and previous cannabis use	UC 33 CD 50
Prospective cohort survey study	Ravikoff and Allegretti et al. 2013 [118]	292	Survey	16.4
Cross-sectional study	Storr et al. 2014 [119]	313	Questionnaire	17.6
Retrospective cohort study	Weiss and Friedenberg 2015 [120]	2,084,895	Survey	67.3
Cross-sectional study	Phatak et al. 2017 [121]	53	Survey	70
Descriptive study	Hoffenberg et al. 2018 [122]	UC: 27 CD: 62 unknown colitis: 10	Questionnaire	32
Cross-sectional study	Benson et al. 2020 [123]	838	Anonymous online survey	25.3

IBD, inflammatory bowel disease; CU, Crohn’s disease; UC, ulcerative colitis.

## Data Availability

Not applicable.
